# Supplementary material for: Predicting the causative pathogen among children with pneumonia using a causal Bayesian network
Source: PLoS Comput Biol. 2023 Mar 13;19(3):e1010967. doi: 10.1371/journal.pcbi.1010967 (PMC10035934; doi:10.1371/journal.pcbi.1010967)
Supplement: Attachment — Submitted filename: Review-v1.pdf [file pcbi.1010967.s004.pdf]

Review of “Predicting the causative pathogen among children with pneumonia using a causal Bayesian network.”

Summary
The paper presents a Bayesian network (BN) model for reasoning about the causes of pneumonia. The model was developed using a combination of expert knowledge and data. The data is 230 cases of pneumonia. The variables in the models are categorised, with some latent variables representing quantities that cannot be directly observed. The use of the latent variables allows the claim that the BN is causal with, in summary, background factors causing the presence of different pathogens, in turn causing the observed signs and symptoms and laboratory test results. The BN is validated inn a number of ways, including ROC and average log loss for some variables with different amounts of evidence, clinical scenarios and sensitivity analysis.

Comments
1. The paper clearly introduces the potential to over-prescribe antibiotics as the clinical problem that is being addressed, noting the potential risk of undertreatment. However, this context / problem is not evident again until section 4.4 and it is not made clear how the BN would be used to help with the decision to use antibiotics, neither which variables would be used nor the acceptable (or achievable) false negative rate for predicting bacterial infection.
2. In line 108, the authors claim that: “BNs provide probabilistic predictions that are transparent and explainable”. It is agreed that relationships between variables in a BN can be displayed in a clearer way than some other modelling techniques, but any claim that this implies explainable predictions needs further justification.
3. It would be help to make clear how the fact that the rate of use of antibiotics in the dataset is 99% does not create a bias. This could perhaps be done by giving more details of the process of collecting the clinical data in relation to the use of antibiotics.
4. In the main text, the number of latent variables is unclear: 9 are mentioned, with 3 shown on Fig. 4. Perhaps the infection variables in Fig 5 are also latent (three are mentioned as such in 3.2.3), but there are 7 infection variables in total. The relationship between the variable class and the method of parameterisation (section 2.2) could also be clearer.
5. It is appreciated that the BN can be used in a flexible way, with no fixed distinction between inputs variable and output variable. Nevertheless, in any clinical decision-making situation for which the BN is proposed as useful, values of one group of variables are observed (potentially with some missing) and posterior probabilities are calculated for others. It is not clear how it is proposed to use the BN. For example, an updated probability distribution for the variable “Clinical diagnosis of bacterial pneumonia” might be useful, but this variable is described as ‘observable’ so why is a prediction needed? If a prediction is needed, is / how is the value present in the dataset (if the parameters are trained in this way) and how confident can we be that recorded value is correct? It may be that these issues can be clarified by further details of the timeline of when the model might be run and when the results of different clinical tests are known.

6. Given the size of some of the CPTs in relation to the dataset size, it would be helpful to explain in more detail how overfitting has been avoided. In particularly, the hybrid expert-data training approach could be explained in more detail with greater clarity on the largest CPTs trained exclusively from data.
7. On line 270, the authors state that “Note that the probability of no pneumonia should always be 0% in our model as it was parameterised using data from a cohort of children who all had X-ray confirmed pneumonia (27).” Please clarify this statement and the purpose of a variable having a state which “should” always have a 0% probability in the model.
8. In Figure 7, is the posterior probability for variable 28 as a different name is used? The figure is hard to read, particularly for input scenario a), perhaps because of a very high proportion of cases having a very low predicted probability. Please find a way to display the data more clearly.
9. The performance of the model is mainly given using log loss (is it an average?) and AUROC. Neither of these metrics address the trade-off between false negatives and false positives, i.e. the sensitivity and specificity in the area where the model might be operated. Further, clarify how the absolute value log loss should be interpreted – what value indicates useful / acceptable performance?
10. The choice of variables in Table 1 could be more clearly justified (and perhaps, for clarity, the variable numbers used) in relation to how the BN will be used. For example, clarify in what circumstance the it is clinically useful to predict “pleural effusion” (the variable is described as observable, in class ‘sign and symptom’). Without this, the AUROC of 0.81 does nothing to evidence the potential clinical usefulness. Additionally, are all the scenarios a) to d) of clinical relevance: if not it is unremarkable that performance improves with more variables observed. It is also not clear why some entries in the table are ‘NA’ (perhaps because I find the sentence “Note for each target and scenario, the variables used to operationally define the evaluation target were removed from the input variables” very unclear).
11. The sensitivity analysis described briefly on pages 11 and 12 is hard to follow. It is not clear which variable (or variables?) are predicted in Figure 8, nor which variables are covered in the three groups (latent concepts, pathogens in the nasopharynx etc) nor what is meant by ‘relevant’ parameters nor how the variation was done.
12. The role of the scenarios in Section 3.4 is unclear. It is stated (line 356) that the scenarios demonstrate potential clinical use, but none of the variables mentioned (the ones used are not completely clear) seem to relate directly to the prediction of a bacterial infection.
13. In line 454, the authors claim “Satisfactory numeric predictive performance has been achieved by the BN in predicting directly observable”. It would be preferable if this statement was further justified by a clear reference to the material in section 3.3.

There are many impressive aspects of this work, including the use of data and expert elicitation, the number and range of experts involved, the importance of the clinical problem and the presentation and description of the BN. However, the progress towards potential clinical application is not sufficiently clear for the work to be of wide.
