# Supplementary material for: Predicting the causative pathogen among children with pneumonia using a causal Bayesian network
Source: PLoS Comput Biol. 2023 Mar 13;19(3):e1010967. doi: 10.1371/journal.pcbi.1010967 (PMC10035934; doi:10.1371/journal.pcbi.1010967)
Supplement: Attachment — Submitted filename: pneumobna_plos comp biol_response to reviewers_v1.pdf [file pcbi.1010967.s005.pdf]

Dear Editor,

We'd like to thank you for the opportunity to revise and resubmit our manuscript to *PLOS Computational Biology*. We'd like to express our gratitude to both reviewers for their positive and constructive feedback.

We have now revised the manuscript by addressing both reviewers' comments point by point as outlined below. By doing this, we have made further clarification around the methodology and interpretation, in particular their implications for clinical decision making. Wording around clinical value has been revised throughout the manuscript, making it clearer about what the work has achieved (shown how this method works and how it would help decision making); what are the next steps (external validation, examining and eventually trialling improvements over existing practices); and what is still a goal (implementation, adoption and practical improvements to antibiotic use). All changes are highlighted in red in the revised manuscript with tracked changes, and all line numbers below also refer to the tracked version.

In addition, we have provided source models, raw evaluation results and python scripts for running our analysis as new appendix and via the Open Science Framework (OSF) (https://osf.io/m97vb/). We did not provide the R file mentioned in the manuscript as it was used to prepare line-listed patient data to the format required for the modelling work, which cannot to be made publicly available due to ethics requirement. Newly provided project data include:

- Source models on OSF:
  - **paediatric_pneumonia_BN_v4.5_priors.dne**; this is the model with prior parameters informed by domain expert knowledge.
  - **paediatric_pneumonia_BN_v4.5_learnt.dne**; this is the model with posterior parameters after training with data (the xdsl version of this file was previously available on OSF).
- Raw evaluation results:
  - **Additional File 3. KL comparison.xlsx**; in this document, we provide the KL divergence assessment results that compare the difference between the prior and posterior CPTs of BN v4.5 (i.e., the two newly provided source models on OSF).
  - **cv_results_pneumonia_v4.5_bac.json** and **cv_results_pneumonia_v4.5_detectNPs.json**; 10-fold cross-validation results used for Table 1 and 2.
  - **fpfn_results_bacOnly.txt**; Table 2 results that compare different preference settings.
- Python scripts:
  - **make_comparison_csv.py**; script for generating the results in **Additional File 3**.
  - **crossvalidations_model_v4.5.py**; script for generating the cross-validation results.
  - **min_fp_fn.py**; script for conducting the Table 2 analysis.

Yours Sincerely,

Yue Wu

On behalf of all authors

Reviewer #1: This manuscript describes a methodology to find causal pathogens for pediatric pneumonia. The study was carried out by surveying multiple doctors (expert knowledge) and use their knowledge along statistical methods to describe a causation DAG for the aforementioned disease which was later tested (AUROC and log-loss) using data obtained from 230 children admitted in a hospital.

The process of constructing the GAN was done iteratively using 3 distinct methods to update the nodes depending on data quality and parametrized via estimation maximization. The nodes consist in 63 total variables of multiple natures (background factors, pathogens, infection/diagnosis, etc). 3 of those variables are latent ones introduced to describe exposure, colonization and progression.

The authors use the bacterial diagnosis as a gold standard as one of the main objectives is the prediction of bacterial pneumonia.

The paper is very well written. The introduction properly states the need of such study (antibiotic usage) and guides through the pneumonia topic extensively and how Bayesian Networks can have an important role in the understanding of causal effects on distinguish bacterial from non-bacterial infections.

The statistical methods were sound, relevant to the study and well described. It's refreshing to have a paper that includes domain experts in the pipeline.

I will recommend for publishing as is but I do have a some questions/comments:

Section 3.3:

"So long as the number of incorrect diagnoses is not great, this will not affect our conclusions" .

What would the authors think the threshold would be?

Nice to have an acknowledgment of the limitations, I appreciate that.

This quoted comment from Section 3.3 refers to the situation where we intend to assess the performance of the BN in predicting the **causative pathogen for pneumonia** (node 25), which, as a latent variable, can never be directly observed, and is thus not possible to test with observational data. Instead, we evaluate the BN's performance in predicting the **clinical diagnosis of bacterial pneumonia** (node 28), which is the current gold standard for operationally defining (therefore a surrogate of) node 25 in clinical practice. In other words, we assume approximately equivalent performance of the BN in predicting node 25 and node 28, based on the explicitly modelled relationship between these two variables in our BN; in brief, node 25 gives rise to **pleural effusion** (node 46) and *positive* **blood culture** (node 53), where in turn nodes 46 and 53 deterministically define node 28 (a clinical diagnosis of bacterial pneumonia would be made if either pleural effusion is diagnosed or bacteria is cultured from blood). The assumption that node 28 is a good surrogate would *not* hold if '*viral-like* pneumonia (node 25)' can also probabilistically lead to pleural effusion or positive blood culture, and in BN v4.5, these probabilities are very low: 3% and 0% respectively. We have now clarified that because the discrepancy is low (less than 5%) in *Section 3.3 (line 375-381)*, this gives us confidence in using this surrogate (node 28) for evaluation purposes.

It appears to me that for the authors the importance of causality is knowing what's the minimum data needed to correctly predict a bacterial pneumonia. It would be nice to have a discussion about the

understanding of the causal effects as a preventive measure or why is it better to have a BN over a fully automated ML algorithm if the goal is the prediction of a labeled variable.

With an ultimate interest in informing health and clinical decision making, we value causal methods (in this case, causal BNs) over other (non-causal) ML algorithms because of their ability to incorporate preventative or interventional effects that act on the modelled (causal) mechanistic pathways. We have now clarified this in *Section 1 (line 130-134)* and further discussed it in *Section 4 (line ~479-482)*. We also further clarified in *Sections 3.3 (line 384-386)* and *3.4 (line 450-451)* that, rather than seeking minimal data input, the input scenarios and clinical scenarios intend to replicate the chronological availability of information in clinical practice as well as the potential time points when BN predictions can be applied for decision support.

As stated in section 4.5 the flexible definition of pneumonia could be problematic although it seems that the important part is the bacterial more than the name itself. Would the scope change if the authors proceed with a larger study proposed?

We agree that the important part is the bacterial attribution more than the variable name itself. More explicitly, it's the identification of bacteria amongst the causative pathogens of pneumonia that's important, given how 'pneumonia' is operationally defined based on data collected from a particular cohort; e.g., a larger study in another country.

To this end, we highlight the important distinction and mapping between variable definitions (or a BN/model dictionary) and a data dictionary. A variable definition (usually) would not change if it's been well designed to describe certain mechanistic process in a problem domain, and can be mapped to an external dataset that intends to capture observations about that very same mechanistic process. Any discrepancy between a variable definition and relevant data definition should lead to a review and potential revision of the model structure with respect to the original modelling purpose (i.e., the scope), with the aim to inform the adaptability of the model, the interpretation of model performance once adapted, and how the BN may be developed for future adaptation.

For example, as stated in the BN dictionary (Additional File 2), **typical bacterial pneumonia** (node 24) is defined as 'typical bacteria is invading the terminal air spaces of the respiratory tract', and all cases in our cohort were x-ray confirmed pneumonia. When applying this model to another dataset where pneumonia maybe more broadly be defined based on clinical signs and symptoms without x-ray evidence, we may consider including an additional site of the lower respiratory tract (e.g., bronchi) in the model and make it clear that 'pneumonia' in the new dataset should be mapped to variables that capture infection of both the terminal air spaces and the bronchi.

We have now further clarified and discussed this in *Section 4.5 (line 583-590)*.

Additional file 1 was important to understand the development of the BN. Might be worthwhile to have in the main paper.

We have now replaced Fig 1 (the simplified schematic of BN development) by the detailed version previously included in the Additional File 1 (*Section 2, Fig 1*). A brief summary of workshop and survey topics has been included in *Section 2.1, line ~176-179.*

Reviewer #2:

Summary

The paper presents a Bayesian network (BN) model for reasoning about the causes of pneumonia. The model was developed using a combination of expert knowledge and data. The data is 230 cases of pneumonia. The variables in the models are categorised, with some latent variables representing quantities that cannot be directly observed. The use of the latent variables allows the claim that the BN is causal with, in summary, background factors causing the presence of different pathogens, in turn causing the observed signs and symptoms and laboratory test results. The BN is validated in a number of ways, including ROC and average log loss for some variables with different amounts of evidence, clinical scenarios and sensitivity analysis.

Comments

1. The paper clearly introduces the potential to over-prescribe antibiotics as the clinical problem that is being addressed, noting the potential risk of undertreatment. However, this context / problem is not evident again until section 4.4 and it is not made clear how the BN would be used to help with the decision to use antibiotics, neither which variables would be used nor the acceptable (or achievable) false negative rate for predicting bacterial infection.

We have now clarified that the key target variable for prediction is the **causative pathogen for pneumonia** (node 25) throughout the manuscript in *Sections 1-4 (lines 144, 168-169, 250-253, 375-377, 408-409, 441-442 and 476)*. This is because from a causal perspective, it would respond (or not respond) to antibiotic use and subsequently drive the progression of disease. As stated in response to Reviewer #1, we also acknowledge that node 25 is a latent variable, which can never be directly observed, and thus cannot be directly evaluated using observational data. Instead, we evaluate the BN's performance in predicting the **clinical diagnosis of bacterial pneumonia** (node 28) as a close surrogate of node 25. A false negative rate (FNR) can only be measured if the target variable is recorded in the test data set, and hence we can only report these for the surrogate (node 28). We have now included additional metrics based on the FNR and FPR for node 28 in *Section 3.3, Table 2*.

We also note that the concept of an 'acceptable' FNR (or FPR) requires one to be explicit about one's preferences (or values) when a decision needs to be made (embedded within some form of utility- or decision-based framework). We wholeheartedly agree this is a useful thing to do – we address this in detail in combination with our response to comment #9 below.

2. In line 108, the authors claim that: "BNs provide probabilistic predictions that are transparent and explainable". It is agreed that relationships between variables in a BN can be displayed in a clearer way than some other modelling techniques, but any claim that this implies explainable predictions needs further justification.

We have now clarified in the introduction (with additional references) that a BN only offers potentially explainable predictions if it was designed to have a causal interpretation. I.e., it models relevant mechanistic pathways that lead to and can be intervened upon to change downstream effects. While not specifically of concern here, Pearl (along with many others) have provided ample arguments for how causal diagrams (including causal BNs) can provide explainable predictions (including identification of the causal impact of individual nodes and pathways). This is not possible to achieve in any non-causal modelling approach, and we've clarified this in the text (*Section 1, line 130-134*), with additional references as listed below.

30. Cooper G. An overview of the representation and discovery of causal relationships using Bayesian networks. Computation, causation, and discovery. 1999; p. 4–62.

31. Pearl J, Russell S. Bayesian Networks. 2000; University of California, Los Angeles.

32. Hernan M, Robins J. Causal Inference: What If.; 2020.

3. It would be help to make clear how the fact that the rate of use of antibiotics in the dataset is 99% does not create a bias. This could perhaps be done by giving more details of the process of collecting the clinical data in relation to the use of antibiotics.

The cohort represents hospitalised children with X-ray confirmed pneumonia. The current standard of care of pneumonia in Australia is to give antibiotics, so we believe the high rate of antibiotics in this population is representative of the target population. We have now further clarified in *Section 4.4 (line 563-565)* that among the 230 children enrolled, 47% were treated with antibiotics prior to hospital presentation when baseline study data were collected, while 99% eventually received antibiotics during their hospital stay (post-enrolment). We believe this pattern of antibiotic use is reflective of the target population (of children hospitalised for X-ray confirmed pneumonia), although this population may not be reflective of the total population of children presenting with lower respiratory infections, many of whom do not attend hospital or have chest X-rays. To this end, as a response to Reviewer 1, we further discussed the ideal process of adapting the model to other cohorts in *Section 4.5 (line 583-590)*.

4. In the main text, the number of latent variables is unclear: 9 are mentioned, with 3 shown on Fig. 4. Perhaps the infection variables in Fig 5 are also latent (three are mentioned as such in 3.2.3), but there are 7 infection variables in total. The relationship between the variable class and the method of parameterisation (section 2.2) could also be clearer.

We have clarified in *Section 3.1 (line 256-262)* that the 9 latent variables include 3 concept variables that describe the epidemiological context (nodes 12-14 in Fig 4) plus 6 infection-relevant variables that describe the sites of respiratory tract and causative pathogens involved (nodes 21-26 in Fig 5). The parameterisation method chosen for each variable was influenced by – but not determined by – their observation status (whether data were available); rather, the choice was made based on the definition of each variable and how well we were able to quantify them based on data and/or expert knowledge. Among all 52 observable variables, 16 used the data-driven method, 34 used the hybrid expert-data method and 2 used the expert-driven method. Detailed parameterisation method used for each variable is provided in the BN dictionary (Additional File 2). This has been further clarified in *Section 2.2, line 201-203 and Section 3.1, line 264-266*.

Note, we've further spotted a typo regarding the total number of observable variables, which should be 52 (rather than 53) where there are 8 (rather than 9) pathogen or infection-relevant variables, 25 signs and symptoms, and 5 lab variables (rather than 24 and 6, with oxygen saturation previously counted as a lab rather than sign variable). We have fixed these numbers in *Section 3.1, line 256-262*. We also fixed one parameterisation method in Additional File 2, from data-only to expert-data for current clinical phenotype (node 52).

5. It is appreciated that the BN can be used in a flexible way, with no fixed distinction between inputs variable and output variable. Nevertheless, in any clinical decision-making situation for which the BN is proposed as useful, values of one group of variables are observed (potentially with some missing) and posterior probabilities are calculated for others. It is not clear how it is proposed to use the BN. For example, an updated probability distribution for the variable "Clinical diagnosis of bacterial pneumonia" might be useful, but this variable is described as 'observable' so why is a prediction needed? If a prediction is needed, is / how is the value present in the dataset (if the parameters are trained in this way) and how confident can we be that recorded value is correct? It may be that these

issues can be clarified by further details of the timeline of when the model might be run and when the results of different clinical tests are known.

We would first like to emphasise the distinction between 'not observed' and 'not observable'. The former refers to the more traditional missing data problem, where certain observations were missed during the data collection process for a pre-defined variable. That is, the variable is observable but may be not observed for certain participants or at certain time points. In contrast, the latter refers to a situation where the variable itself describes something that can never (in practice) be directly observed, e.g., the replication process of a virus inside a patient's lung. Our variable classification with respect to the observation status refers to the latter, we classify each variable in the BN as 'observable' or 'not observable (i.e., latent)'. As stated in response to Comment #1 above, the latent variable **causative pathogen for pneumonia** (node 25) is our primary target for prediction to aid diagnostic support and can subsequently inform the choice of antibiotic use potentially depending on a utility-based assessment (which is beyond the scope of this current work).

Given the flexibility of BNs, prediction on node 25 can be made with any input information depending on what may be available to the end-user at any timepoint when decision support is required (even with no input where the model will provide a marginal prediction based on the eligible cohort). The variable **clinical diagnosis of bacterial pneumonia** (node 28) is by definition an *observable* variable (because we can collect data on it) but would often be *not observed* at the point when a diagnostic decision is needed, and therefore would not be entered as a model input to generate a prediction for node 25.

We believe we have already addressed the question of "*when the model might be run",* but could perhaps have further clarified, which we do in *Section 3.3 (line 384-386)*. The four input scenarios a-d intend to approximately replicate how various information may become available chronologically in clinical practice, and are also potential time points when the BN might be used for decision support. We do not claim the BN *should* be used at all of these time points; rather we explore what the value might be of using the BN at each such point. Of note, node 28, as well as its operational definition pleural effusion and positive blood result, are all excluded from these scenarios (i.e., we assume they would not be available when the BN is applied for decision support).

As briefly alluded to in Sections 4.4, much more work is required to translate a mathematical model (however well-validated) into a useful tool that aids decision support in practice. This requires a user-interface (UI) which presents model outputs in a form that end-users can understand, interpret and use – implying that users have an understanding of the scope of the underlying model and how it relates to information they are to enter as input. For example, research is needed to understand how a UI can help facilitate high quality data entry. In *Section 4.5 (line 583-590)*, we have further elaborated on what data should be used in the context of future evaluation, adaptation and interpretation of the model.

6. Given the size of some of the CPTs in relation to the dataset size, it would be helpful to explain in more detail how overfitting has been avoided. In particularly, the hybrid expert-data training approach could be explained in more detail with greater clarity on the largest CPTs trained exclusively from data.

Several measures were taken to address the overfitting problem. Firstly, all evaluations made use of a 10-fold cross-validation, demonstrating stable model performance regardless of which subset of the cohort dataset was used (the full evaluation results for this analysis are now made available via OSF). Secondly, as shown in the updated Fig 1 (in response to a comment from Reviewer #1), the model structure has been revised iteratively to achieve its current status (v4.5) which included several rounds of simplification to reduce CPTs sizes (e.g., by introducing intermediate nodes). As a result, as

detailed in Additional File 2, all 16 CPTs that were exclusively trained from data have no more than three parent nodes (mostly two), and no more than three states (mostly binary). This leads to CPTs consisting of a maximum of 81 cells, i.e., 3-4 data points per cell in the worst case (with an average around 15). There are 7 nodes parameterised exclusively using expert knowledge (which, by definition, cannot be overfitted, at least not in any traditional sense). And for the hybrid expert-data (constituting the majority of the variables), the expert-informed priors were assigned with weight of one data point and subsequently updated as part of the full model via EM; for which we have now further clarified in both *Section 2.2 (line 197)* and *Section 3.1 (line 264-266)*. The weight of 1 on the expert-informed priors is sufficient to mitigate overfitting when there is very little data (e.g. only 2-3 cases). It is still possible for overfitting to occur with a larger but still small number of cases (e.g. 15-20 cases), but for such numbers, the cross-validation will have enough data to be able to both evaluate the model as well as penalise any overfitting.

In addition, while acknowledging that overfitting cannot be properly determined until we can assess the model using an external dataset, it is both possible and (we now believe) important to identify which parts of the model are most closely fitted to our cohort. This would allow these parts of the model to be a focus of the evaluation when applying or evaluating the model on external datasets. To this end, we have adopted an approach that compares CPTs between the trained model and prior model using Kullback–Leibler divergence, highlighting in particular the discrepancies between prior and posterior CPTs among the hybrid expert-data variables, i.e., which expert-informed CPTs were shifted the most by the data. With full results attached now as *Additional File 3*, we noticed that both viral-like pneumonia (node 23) and typical bacterial pneumonia (node 24) rank highly among the hybrid expert-data variables. We have updated the manuscript with this new analysis in *Section 2.3 (line 223-226)*, *Section 3.3 (line 431-438)*, and discussed overfitting in *Section 4.5 (line~590-595)*.

7. On line 270, the authors state that "Note that the probability of no pneumonia should always be 0% in our model as it was parameterised using data from a cohort of children who all had X-ray confirmed pneumonia (27)." Please clarify this statement and the purpose of a variable having a state which "should" always have a 0% probability in the model.

The state 'no pneumonia' was introduced to ensure node 25 'causative pathogen for pneumonia' can be well-defined when there are '*no* typical bacterial pneumonia' and '*no* viral-like pneumonia'. Therefore, the 'no pneumonia' state is essentially required to have a sound model structure, but has no corresponding data in our cohort (i.e., cases with X-ray confirmed pneumonia). We have assumed all cases in the cohort are either typical bacterial or viral-like pneumonia. However, this assumption is imperfect: there may be (a low but non-zero chance of) mis-classification of 'pneumonia' due to potential limitations of the X-ray assessment.

We used the EM algorithm for this (latent) node – which in this case, can be very approximately considered to produce an unsupervised classification or clustering. EM will produce as many 'classes' or 'clusters' as it is given states to parameterise. Here, EM's parameterisation of node 25 into 'typical bacterial / viral-like / no pneumonia' resulted in approximately 7% of cases being classified (or clustered) into the 'no pneumonia' category – ultimately suggesting these 7% of cases look more like each other, than they do 'typical bacterial' or 'viral-like' cases.

Ideally, we would like to improve the classification by explicitly representing a more comprehensive spectrum of lower respiratory tract infections (for example, including bronchitis). This requires us to determine how they should be explicitly mapped to other model variables, but this can only be achieved with access to relevant data. In the absence of such data, by setting 'no pneumonia' to 0% (i.e., X-ray confirmed pneumonia node 27 to 100% 'case'), we force the model to re-classify those

7% of cases as either ‘typical bacterial’ or ‘viral-like’ – which is still valuable for the application purpose of guiding antibiotic use.

We have now further clarified the above in *Section 3.2.2* (*line 324-328*) and *Section 4.5* (*line 606-610*).

8. In Figure 7, is the posterior probability for variable 28 as a different name is used? The figure is hard to read, particularly for input scenario a), perhaps because of a very high proportion of cases having a very low predicted probability. Please find a way to display the data more clearly.

The variable name for node 28 has now been made consistent in *Section 3.3 (line 383 and Fig 7)*, and the layout of Fig 7 has been revised with improved readability.

9. The performance of the model is mainly given using log loss (is it an average?) and AUROC. Neither of these metrics address the trade-off between false negatives and false positives, i.e. the sensitivity and specificity in the area where the model might be operated. Further, clarify how the absolute value log loss should be interpreted – what value indicates useful / acceptable performance?

As described in Section 2.3, the log loss is considered more informative as it rewards the accuracy of probability estimates against data. Therefore, we used its mean as a way to understand when models offer probabilistic predictions that fit the frequencies observed in the data better.

To quantitatively evaluate the model predictions from a clinical decision perspective, we agree with the reviewer that a useful model should explicitly explore the trade-off between false negatives and false positives. We propose further that the trade-off should be defined under particular decision-making contexts, e.g., how much clinicians are willing to accept false positives (over-treat a non-bacterial infection with antibiotics) in order to avoid false negatives (missing/delaying antibiotic therapy for a bacterial infection). This ultimately hinges on the *values* (or utilities or preferences) that one attaches to the consequences of each type of error. As noted, we wholeheartedly agree that being explicit about how these values impact the use of any model is critical, and thank the reviewer for encouraging us to investigate this here.

We have now included *Table 2 in Section 3.3* to illustrate variations on this trade-off, where we present the preferred threshold for achieving optimal model performance in predicting the clinical diagnosis of bacterial pneumonia (node 28), with associated false negatives (FN), false positives (FP), sensitivity and specificity in predicting the clinical diagnosis of bacterial pneumonia (node 28). In particular, we define *optimal* based on a minimised weighted sum of the false positive and false negative rates (FPR and FNR) across the 10-fold cross-validation results. We show that the optimal threshold can be very sensitive under different input scenarios and preference settings including where P1) the false positive rate and false negative rate are equally weighted; P2) the false negative rate has a weight that is three times that of the false positive rate; P3) the false negative rate has a weight that is five times that of the false positive rate; and P4) the false negative rate has a weight that is 10 times that of the false positive rate.

We have revised several sections of the manuscript to integrate this new analysis and discuss its implications, including the *Abstract, Section 2.3 (line 212-214)*, *Section 3.3 (line 414-422, Table 2)* and *Section 4.4 (line ~550-559)*. Both the raw evaluation results and script for generating these results are now made available via the open science framework.

10. The choice of variables in Table 1 could be more clearly justified (and perhaps, for clarity, the variable numbers used) in relation to how the BN will be used. For example, clarify in what circumstance the it is clinically useful to predict "pleural effusion" (the variable is described as observable, in class 'sign and symptom'). Without this, the AUROC of 0.81 does nothing to evidence the potential clinical usefulness. Additionally, are all the scenarios a) to d) of clinical relevance: if not it is unremarkable that performance improves with more variables observed. It is also not clear why some entries in the table are 'NA' (perhaps because I find the sentence "Note for each target and scenario, the variables used to operationally define the evaluation target were removed from the input variables" very unclear).

The primary purpose of *Section 3.3* is to estimate how well the model can predict the target (node 25). This target is latent, so we instead evaluate several surrogate (and closely related) observable variables. Even though these observable variables might not be routinely available at the time the BN would be used, they are of course always available in our test set. The surrogates include node 28 as mentioned before, two variables that define node 28 (pleural effusion, node 46, and positive blood culture, node 53), as well as five pathogen specific variables that describe their presence in the nasopharynx. These are now made clear in *Table 1*. We expected to see improved performance in predicting these observable variables as more variables were observed as a way of confirming the model would behave in a reasonable way when predicting node 25.

Although it's not our primary purpose to demonstrate clinical usefulness, the four input scenarios in Table 1 (scenarios a-d) are still indicative to some extent of 1) real-world clinical decision points and 2) the information available at such decision points. Understanding what level of performance the model is able to achieve given data availability along such a timeline is helpful in assessing the potential usefulness of the model in practice. As described in the above response to comment #9, we expect the newly added analysis about utility preferences (Table 2 in particular) also helps to better address questions around clinical usefulness and potential implications for decision making.

The 'NA entries' in the table mean that the prediction targets themselves were included as observed variables in the corresponding input scenario. There is no point reporting the accuracy for these variables in these cases, as the 'answer' is provided to the model. Hence the 'NA'. This was what the quoted note was intended to convey.

We have now revised the manuscript to make these points clearer (*Section 3.3, line 408-413 and Table 1*).

11. The sensitivity analysis described briefly on pages 11 and 12 is hard to follow. It is not clear which variable (or variables?) are predicted in Figure 8, nor which variables are covered in the three groups (latent concepts, pathogens in the nasopharynx etc) nor what is meant by 'relevant' parameters nor how the variation was done.

As described in *Section 2.3*, Variance-based sensitivity analysis (VBSA) was used for the sensitivity analysis. In particular we varied the CPTs of a group of variables simultaneously and observe how the distribution of a target variable state spread given the varying CPTs. We have clarified this further in *Section 2.3 (line 217-222)* and *Section 3.3 (line 423-430)* and provided details on variables involved in the sensitivity analysis in *Fig 8*.

12. The role of the scenarios in Section 3.4 is unclear. It is stated (line 356) that the scenarios demonstrate potential clinical use, but none of the variables mentioned (the ones used are not completely clear) seem to relate directly to the prediction of a bacterial infection.

Section 3.4 was designed to illustrate the clinical usefulness from a qualitative perspective, presenting raw probabilistic predictions of the (latent) primary target node 25 (causative pathogen for pneumonia). Proposed and reviewed by domain experts, selected variables in these scenarios are not those you would specifically measure or look for when suspecting bacterial pneumonia – rather these are information commonly presented where clinicians need to make a diagnosis of bacterial pneumonia and decide on interventions such as antibiotic use. All the entered variables are still predictive for typical bacterial pneumonia because they sit on relevant causal pathways as described by the BN. We have revised the *Section 3.4 (line 443-446)* to make this clearer and included the node name and number of each variable mentioned in the scenarios (*Fig 9-11*).

13. In line 454, the authors claim "Satisfactory numeric predictive performance has been achieved by the BN in predicting directly observable". It would be preferable if this statement was further justified by a clear reference to the material in section 3.3.

We have now referred to example metrics reported in *Section 3.3* and further discussed what they imply for achieving the goal of a decision support tool that improves patient management and encourages judicious use of antibiotics for paediatric pneumonia (*Section 4.4, line 550-559*).

There are many impressive aspects of this work, including the use of data and expert elicitation, the number and range of experts involved, the importance of the clinical problem and the presentation and description of the BN. However, the progress towards potential clinical application is not sufficiently clear for the work to be of wide.

Having gone through significant revision by addressing each comment from both reviewers, we believe the manuscript has now clearly described what the work has achieved (shown how this method works and how it would help decision making); what are the next steps (external validation, examining and eventually trialling improvements over existing practices); and what is still a goal (implementation, adoption and practical improvements to antibiotic use).
