# Supplementary material for: Predicting the causative pathogen among children with pneumonia using a causal Bayesian network
Source: PLoS Comput Biol. 2023 Mar 13;19(3):e1010967. doi: 10.1371/journal.pcbi.1010967 (PMC10035934; doi:10.1371/journal.pcbi.1010967)
Supplement: Attachment — Submitted filename: pneumobna_plos comp biol_response to reviewers 2_v1.pdf [file pcbi.1010967.s006.pdf]

Dear Editor,

Thank you for the opportunity to revise and resubmit our manuscript to *PLOS Computational Biology*. We have now revised the manuscript by addressing the review recommendations point by point as outlined below. In the Acknowledgement section, we further thanked anonymous reviewers for their valuable comments and suggestions. All changes are highlighted in red in the revised manuscript with tracked changes, and all line numbers below also refer to the tracked version.

We look forward to hearing from you.

Yours Sincerely,
Yue Wu
On behalf of all authors

Reviewer #2:

1. I am grateful to the authors for their detailed response to my comments and the updates to the paper.

2. Table 2 is a great addition to the paper. I note that the sensitivity and specificity are given as rates whereas the false positives and false negatives are numbers of cases. A consistent approach (percentages perhaps) would be preferable. Otherwise, please at least mention the dataset size (230 I think) so that the numbers can be interpreted.

Table 2 in Section 3.3 (*pg 12*) has now been updated. Both false positives and false negatives are presented in percentages, and the dataset size has been further clarified in the caption.

3. I appreciate the new discussion in section 4.4 about the trade-off between FPR and FNR, but is it possible to be a little less abstract? My (possibly simplistic) interpretation of the clinical goal to identify true negatives (reduce unnecessary antibiotic use) without false negatives (potentially endangering patients). Looking at Table 2 on this basis, with the clinical data corresponding to column (a), the predictor is roughly useless, as 202/205 are false positives, matching current clinical practice. On the other hand, row P3 and column (d) shows the predictor avoiding approx. 40% of the unnecessary uses of antibiotics, which is potentially of benefit. I guess there may be circumstances (patients carefully observed?) in which a higher FNR is acceptable but these could be mentioned.

We would still appreciate some level of abstraction here, as it is useful when generalising the concept to other clinical problems and decision-making problems in general. We've re-organised the Section 4.4 to improve the flow of text, where we introduced the concrete examples suggested by the reviewer (Section 4.4 *line 544-591*), and further highlighted the key message from this section at the beginning of the Discussion (Section 4, *line 479-481*).

For clarity, we have revised our nomenclature throughout the manuscript from 'clinically-diagnosed' to 'clinically-confirmed' bacterial pneumonia, in acknowledgement that some children without highly probable bacterial infection (clinically-confirmed) were nonetheless suspected of having bacterial infection. Wherever 'preference setting' is referred in the manuscript, we have also clarified it means the 'FPR/FNR trade-off preference'.

4. The first paragraph of the discussion section still contains the statement that 'the model performed well'. I do not think this very general statement is justified. For example, fig 7 (d) shows that two positive cases were given very low predicted probabilities even in the input scenario with most data; in table 1 the AUROC ranges from <0.5 to 0.8. Given all the detailed attention to characterising the performance of the model (particularly in section 4.4), please could this unqualified statement be amended or removed?

The statement has been removed, and the sentence was amended as 'We used AUROC and log loss to quantitatively assess the model's performance in predicting the directly observable variables like the diagnosis of pleural effusion, positive blood culture and detection of RSV and mycoplasma in the nasopharynx. Based on 10-fold cross-validation, the model performance steadily improved as more information was entered (Table 1)' (Section 4, *line 475-478*)
